# Medical and Physical Intelligence

**Published:** 1826-05

**Authors:** 


### MEDICAL AND PHYSICAL INTELLIGENCE.

### PHYSIOLOGY.

1. *Animal Magnetism.* — THIS seems again about to occupy the attention of the French medical school. At a late meeting of the Academie Royale de Medecine, M. HUSSON, in his own name and that of several other physicians, read a Report on the question, Whether the section should again consider the subject of animal magnetism? The commission concludes affirmatively,—1st. That the judgment given in 1784, by the members of the Academy of Sciences, and of the Royal Society of Medicine, charged with the examination of the subject of animal magnetism, ought not to interdict a fresh examination; since, in matters of science, a first judgment has been too often found defective, and because the searches made by them had not been made with all the care that the habit of experimenting has now introduced in the exposition of facts.

2d. That the magnetism on which judgment was pronounced in 1784, differs entirely, both in theory and practice, and its phenomena, from that which is now to be considered.

3d. That magnetism, having now fallen into the hands of learned men and physicians, and being a special subject of study in most of the colleges of medicine in other countries of Europe, it is for the honour of French physicians not to be behind those of other nations. In fact, that, in considering magnetism as a secret remedy, it is not only an amusement, but a duty of the Academy to take notice of it.

The discussion on this interesting subject has been postponed for some future meeting.

M. I. AMADEE DUPAU has just published a work relating to this subject, "Lettres Physiologiques et Morales sur le Magnetisme Animal," in which there is much interesting matter. He considers the moral influence of the magnetism to be very great over his patients, and that much danger is to be apprehended, both to the public morals and the safety of private families.

One of the advocates for the system, M. ROSTAN, speaking of the influence acquired by the operator over the patient, says, "that she would follow him as a dog follows his master." If this be true, it is high time for fathers, husbands, and brothers, to look about them.

2. *Uterus wanting.*—M. RENAUDIN presented to the Academy of Medicine the genital organs of a woman, in whom the uterus was wanting. This woman, fifty-two years of age, died of a cancerous affection of the stomach; she was of very small size, not more than three and a half feet in height; her intellects imperfect; and she had never menstruated, neither had her breasts ever been developed. The parts of generation externally were well formed; the hymen existed in part; and a finger, introduced into the vagina, encountered, instead of the neck of the uterus, a small tubercle, possessed of little sensibility. Between the bladder and rectum, instead of a uterus, was a kind of firm cord, about the size of a quill, communicating at one extremity with the vagina, and also with the fallopian tubes. These tubes, very much enlarged at the point where they opened into this canal, formed there a kind of sac. Some traces of ovaria were faintly perceptible. On slitting open the vagina and this little canal, the first was found to be properly formed, and the last, which was only an inch in length, was evidently, both from its consistence and organisation, the neck of the uterus imperfectly formed; the body and fundus of that organ being altogether wanting. (*Archives Generales*, Mars.)

---

### PATHOLOGY.

3. *Aneurism of the Aorta.*—Wm. Simpson, aged thirty-seven, a man much addicted to drinking, a private in the Coldstream Guards, was admitted into the regimental hospital on the 13th instant. He was evidently very ill, and had been suffering for several weeks with great difficulty of breathing and bad cough. Early in the night before his admission, he began to spit blood, and continued to do so all night, occasionally in considerable quantity. He was coughing, and spitting up large mouthsful of blood at this time, and complained of being very weak and faint. The pulse was labouring and oppressed, and a large quantity of blood was therefore taken from his arm. He took ol. ricini; was ordered a blister to the chest, and two grains of the acetate of lead, made into a pill with the conf. opii, every three hours. In the evening, he said that he was rather easier, and his cough was less troublesome. The following night was passed badly; he had been sick, and vomited, and felt very ill; he was restless and anxious, but did not complain of suffering pain or uneasiness about the chest, but only of a stoppage at his throat, which made his breathing difficult; his cough was very frequent, and he still continued to spit blood. About two o'clock P.M. he vomited largely; about half an hour after which, in again straining to vomit, he threw up about a pint of blood, sunk backwards in his bed, and expired.

The body was examined on the 16th, and the following appearances presented themselves:—On raising the sternum, both lungs appeared elevated and protruding forwards, yet they were in appearance healthy; that of the left side was quite sound. The right side of the thorax was full of blood, in part coagulated and partly fluid: the quantity amounted to five or six pints. This was carefully removed, and the surface of the lung examined, in order to discover the breach through which the hemorrhage had taken place. At the back part of the lower lobe, a round opening, large enough to admit the passage of a half-crown piece, was discovered, and a grey-coloured coagulum lay in the opening. Under the impression that all the mischief was confined to the lung, it was about to be removed; but, on cutting between it and the spine, to detach it at the root, a cavity was laid open leading to the left, and in it the same grey substance which appeared through the opening in the lung. Passing the finger within this cavity, four or five carious vertebræ were felt; it then became apparent that this was an aneurism.

The heart was next examined: it was healthy, and rather a small one.

The quantity of serum in the pericardium was greater than natural. The finger was then passed through the cavity into the arch of the aorta up to the heart; next dividing the aorta below the diaphragm, it was dissected upward. The dilatation lay about half-way between the diaphragm and the curvature, occupying the space of four vertebræ in length. The vessel was quite membranous. The aneurismal sac was formed of the aorta, the spine, the pleura of the right side, and a portion of the inferior lobe of the right lung. The vertebræ were carious; the pleura was thickened almost to cartilage, and the lung hepatised. The coagulum of the aneurismal sac was shaped like a tongue, the apex and body lying on the spine; the thicker and more irregularly shaped part, corresponding to the root, was contained in the cavity formed within the substance of the lung: the interior of this was laminated. The substance of the lung being the weakest part of the boundary of the sac, was the first to yield to the impetus, and gave way finally into the cavity of the thorax. (*Private Letter from* Mr. MAYNARD, Surgeon, Coldstream Guards.)

4. *Disease of the Lungs.*—At a meeting of the Academie Royale de Medicine, M. LAENNEC presented a lung, which showed some important alterations. It was taken from a man, aged seventy-two, who died from an hæmoptysis of nine months' duration, and which had been in vain opposed by bleeding, purgatives, white oxyde of antimony, blisters, &c. During life, percussion of the thorax had given a flat sound on the right side and behind; respiration did not reach that place. A sonorous rale, on the contrary, was heard in the larger bronchiæ. The patient died from weakness, always increasing. On opening the body, two pounds of serum was found in the right side of the thorax; the lung of that side adhered to the pleura by organised bands, and showed on its surface many schirrous granulations. Its tissue exhibited the alteration known by the name of grey hepatisation, and, scattered here and there, several hæmoptoic points. Many of the branches of the bronchiæ of this lung were evidently infiltrated with that kind of accidental production called cerebriform matter. The heart, although not altered, was covered on its anterior face with similar cerebriform matter, forming a coat nearly two lines in thickness, and sprinkled over with little vessels. The right division of the pulmonary artery presented an ancient fibrinous concretion, which adhered firmly to the coats of the vessel. (*Revue Medicale.*)

5. *Neuralgia from a Wound.*—M. LISFRANC relates the following case:—A man had been struck by a fusil on the sinciput. From this simple contusion of the soft parts, a violent and permanent pain had resulted in the part which had been stricken, with an extreme sensibility of the organ of vision, with other symptoms, both local and general, characteristic of the above complaint. M. Lisfranc removed, by two semicircular incisions, that portion of the integuments which was the seat of the pain; and, by causing the wound to suppurate, effected the cure. M. GEMELLE adds, that, in several analogous cases at the military hospital of Gros Caillou, he succeeded in removing the symptoms by a simple incision down to the bone, which he permitted to suppurate. *(Archives Generales.)*

6. *Bones containing Metallic Mercury.*—These were found in two subjects, both young. In the first, the ribs, the ossa ilium, the upper and lower bones of the leg, exhibited this appearance on being struck; and at present, several years after, mercury can still be shaken from them in the same way. The metallic mercury flowed out upon the dissecting tables, macerating tubs, and other apparatus employed by Professor OTTO, and his friend Dr. GURET, whilst in the act of cleaning the bones. A piece, taken from the middle of one of the bones, was analysed by Professor FISCHER, and demonstrated evidently the presence of mercury. In fine, Dr. Otto has published these two singular facts, as affording "the most undeniable evidence of what is so generally doubted, the power of the living system to reduce the oxides of mercury, and the actual appearance of the latter, so reduced, in the bones." (*Edinburgh Journal of Medical Science*, No. 2.)

7. *Hydrophobia in the Horse.*—I had yesterday (April 25th,) an opportunity of witnessing hydrophobia as it occurs in the horse. The following are the particulars:—

Five weeks ago, a stable dog at Mr. Anderson's bit a coachman, who immediately had the parts cut out and cauterised. Having observed, during the night, a great "to do" between the dog and horse, a *regular practitioner* in this department of the healing art being called in, declared the dog to be rabid, and he was destroyed. The horse, which was of a remarkably quiet and docile disposition, did not appear to suffer any inconvenience, till a few days ago, when he became dull; and in the evening of the 24th, he was bled. Next morning, Mr. Bowtall, a respectable veterinary surgeon, was sent for; who informs me that he found the horse very nervous, with a small weak pulse, about forty, (the natural pulse of the horse is from forty to forty-five.) The horse was again bled, and a laxative ball was administered, together with some "fever medicine." He was then removed to Mr. Bowtall's Infirmary.

During the day, his symptoms continued to increase rapidly. On an empty pail being placed before him, and some water poured into it, he became affected with convulsions of the jaws and throat, but afterwards drank a considerable quantity. One of the earliest symptoms of illness had consisted in an entire change of disposition, so that he now rushed at any one who went near him; and his groom, trusting to the horse knowing him, and thus venturing within his reach, had nearly been bitten. Between six and seven, when I saw him, he was breathing very hard, and seemed much exhausted. His head appeared to be pulled down towards his chest by a frequent nodding motion, which seemed to be convulsive; saliva, tinged with blood, flowed copiously from his mouth; his eye was wild, and his whole appearance expressive of great anguish and ferocity. He was placed in a part of the stable separated from the rest by a partition, which was surmounted by an iron railing: he remained for a few minutes without taking any notice, till a groom, who stood by me, called to him, when he turned suddenly round, making a plunge at us, and dashing his head through between the bars with such violence as to bend them, and cover my hand, at which he snapped, with saliva. After this, or any similar exertion, he fell, and remained unable to rise for some time after.

He was killed in the evening; and, on opening the body this morning, no morbid appearance was discovered, except a slight degree of inflammation about the heart. R. M'C.

*April 26.*

---

### PRACTICAL MEDICINE.

8. *On Bleeding in Pulmonary Inflammation.*—In a short Essay on Bleeding in Pulmonary Inflammations, by M. DUCASSE, fils, after regretting that some men, highly esteemed in the profession, had renounced general bleeding, and trusted entirely to the local application of leeches, (which M. Ducasse thinks most inefficacious in the treatment of active inflammations,) he goes on to prove, by reasoning, the necessity of general bleeding to accomplish the ends desired in treating inflammation, and which ends, he thinks, cannot be accomplished by leeches. In support of his opinions, he cites some cases where the effects of general bleeding are decided, and he sums up his reflections by proposing four rules.

1st. That, in pulmonary inflammations, the evacuation of blood is the most efficacious remedy.

2d. That this evacuation, to do good, must be abundant and repeated.

3d. That the bleeding by opening veins, is that which should chiefly be used.

4th. That leeches can never take place of venesection, and ought only to be used as secondaries in the treatment of inflammation.

These rules are well known and acted upon in this country; but in France, where this decided mode of treating inflammatory complaints is not by any means generally adopted, they cannot be too often repeated. *(Revue Medicale.)* Blood enough, old [illegible]!

9. *Case of a Child, ten months old, who took twenty-four grains of Calomel at two doses.*—Mary Ann Brannan, a female child, ten months old, was brought to me on Tuesday, March 14. She had what might be called a *hydrocephalic* expression, lying in the mother's arms, with the head thrown back; with lack-lustre eyes, strabismus, frequent convulsive twitchings of the face, and in that general state of stupor and abandonment so characteristic of this disease. I remarked to the pupils that it was probably a case of hydrocephalus, and then proceeded to examine more minutely the state of the symptoms. I found that the child had been apparently well till a week before, when it had been seized with fits of screaming, the cause of which was not apparent, as there had been neither derangement of the bowels, nor other obvious indisposition, to account for the occurrence. It was now also observed that the child shut its eyes when the face was turned towards the light, and that it could not be induced to take notice of any thing, although it had formerly been lively. This state appears rapidly to have passed into coma, as the mother said the child had slept nearly the whole week, except when affected with a paroxysm of screaming. At present, in addition to the above-mentioned appearance of countenance, &c. I find the pupil is contracted, remaining in the same state when placed in the shade, and when exposed to bright daylight or to that of a candle: she obviously dislikes the light, as the experiment excited some stifled screams, and increased the convulsive movements of the face.

Two grains of calomel were ordered every two hours. Twelve such powders to be sent.

16th.—The mother having neglected to give her address, the patient was not seen yesterday. The calomel ordered on the 14th, instead of being divided into separate powders, was inadvertently given in one, containing the whole twenty-four grains. The parents having procured this at seven in the evening, and supposing it intended for two doses, gave half of it at that time, and the other half about two hours after. Some, but not considerable, vomiting followed the first dose; and the second was followed by vomiting still more slight: whether any (and if any, what quantity,) of the calomel may have been thus ejected, it is impossible to ascertain. Some hours afterwards, the child began to pass copious green stools, which the mother compared to "chopped parsley and water." It is conjectured by the parents that a dozen such evacuations took place in the course of Tuesday night and Wednesday. Towards yesterday afternoon the purging subsided, and the last motion was passed this morning at two o'clock: it is said to have been costive. During the operation of the medicine, the child appeared much exhausted, but seemed more alive to pain than it had been since the first days of its illness, crying in a natural manner, and appearing to the attendants to be griped.

At present the child notices what is passing around it, following with its eye any one who moves; the expression of the face is natural; the squinting is gone; the pupil contracts on turning it towards the light, and again dilates in the shade. There is no appearance whatever of coma or convulsions. It has not taken the breast this morning: during the night, and ever since the calomel was administered, the milk, and every thing else (as thin panado), has been vomited.

To have two tablespoonsful of lime-water, with an equal quantity of milk, three or four times a-day.

17th.—Medicine retained. Has slept quietly, although not longer than half an hour at a time during the night. Has taken the breast well, and without subsequent vomiting; and has passed two natural stools.—To be put into a warm bath to-night, and to continue the lime-water.

26th.—The child has had no symptom worthy of notice since last report, and seems now to be quite well. R. M'L.

### STATISTICAL MEDICINE.

10. *Annual Report of the National Vaccine Board to the Secretary of State for the Home Department.*

To the Right Honourable ROBERT PEEL, Secretary of State, &c. &c.

SIR,—According to the Bills of Mortality for 1825, the deaths by small-pox amount to 1,299; a much greater number than has been reported for some years past. We had reason to apprehend, from our communications with medical practitioners in various parts of the country, that this disease had prevailed lately with more than its usual malignity; and our suspicions have been confirmed by what has occurred in the metropolis.

From this melancholy statement, it is impossible to avoid the conclusion that, although during the last year 2000 more persons have been vaccinated by our stationary vaccinators, than during any former year, yet the lower orders of society continue to be prejudiced against vaccination, and so careless of the issue, that they still allow small-pox to take its course.

And yet what argument more powerful can be urged in favour of vaccination, than the daily remark which the least observant must make, that, in our churches, our theatres, and in every large assemblage of the people, to see a young person bearing the marks of small-pox is now of extremely rare occurrence?

To what can the freedom from the vestiges of so loathsome a disease be attributed, but to the protecting influence of vaccination? for inoculation has now been disused by all respectable practitioners for some time past.

That a considerable number of persons have had small-pox after having been vaccinated, we are ready to admit; although, of cases of this kind presented to us, a large majority are found, on examination, to be without that test of the operation having been performed successfully and effectually, which all agree to be necessary to perfect security; yet some, from a peculiarity of constitution, similar to that, perhaps, which admits the small-pox twice, are still susceptible of the variolous infection.

But we do, at the same time, continue to contend, on the fullest evidence, that the subsequent disease is a safe one, and frequently as mild as the chicken-pox; which, when it occurred, as it often did after inoculation, occasioned neither alarm nor surprise.

Vaccination, therefore, it will be said, does not afford an absolute and perfect security. We do not present it to the world with that pretension; but we declare that it is the least imperfect of the resources which we possess,—that it has as many advantages over inoculation (which we desire it should supersede,) as the latter has over the natural small pox; besides this great and peculiar merit, that it communicates no contagion: for it should be remembered that inoculation, wheresoever it is used, there it establishes immediately a source of infection; and it is notorious that, whatever protection individuals might experience from it, the mortality in London was eventually increased by it, as it was the occasion of keeping up a constant supply of contagious disease.

We continue to receive applications from all quarters of the world for vaccine lymph; and, in answer to our correspondents, never fail to communicate to them such improvements in the management of vaccination, as our experience may from time to time suggest. That the success of this great resource depends very much on the manner in which the process is conducted, is proved by our own observations in this country, and is amply confirmed by the accounts which we receive from the continent; in many parts of which, where the method inculcated by this Board has been adopted, the small-pox may be said to be almost, if not altogether, extirpated.

*(Signed by the usual Officers.)*

11. *Particulars of the twelve fatal Cases of Small-Pox subsequent to presumed Vaccination, which occurred at the Small-Pox Hospital in* 1825.

| *No.* | *Name.* | *Age.* | *When and where Vaccinated.* | *Character of Cicatrix.** |
|---|---|---|---|---|
| 1 | John Richardson | 26 | in the country | not perceptible. |
| 2 | Samuel Lacey | 25 | ditto | small. |
| 3 | Sarah Woof | 23 | ditto | ditto. |
| 4 | Robert Hanson | 25 | reports himself to have been vaccinated | not perceptible. |
| 5 | John Tubb | 27 | in Hampshire, at ten years of age | ditto. |
| 6 | Eliza Olney | 18 | at Hitchin, Herefordshire | large and smooth. |
| 7 | Stephen Booth | 22 | in Cheshire | large and long. |
| 8 | Thomas Cater | 25 | in Worcestershire | large. |
| 9 | William Johnson | 27 | in Bedfordshire | small, not indented. |
| 10 | W. Wells | 19 | in the country | like a large burn scar. |
| 11 | Henry Smith | 22 | in Suffolk | could not be ascertained. |
| 12 | Mary Butler | 23 | in Wiltshire | hardly perceptible. |

12. *Letter from* Sir HENRY HALFORD, *President of the Board of the National Vaccine Establishment, to* HENRY HOBHOUSE, Esq. *Under Secretary of State, &c. &c. &c.*

SIR,—In obedience to the orders of Mr. Secretary Peel, the Board of the National Vaccine Establishment proceeded, without delay, to consider the Report of the Physician of the Small-Pox Hospital. The only part of that Report which seemed important, was that which stated that twelve persons had died of small-pox, in the Small-Pox Hospital, after vaccination.

* The following are the characters of a perfect cicatrix:—It should be distinctly defined, perfectly circular, indented, radiated, and not larger than the size of a common wafer. The diameter of the scar, however, is of less importance than its circular and well-defined edge.

To authenticate this fact, if it were a fact, the Board requested the attendance of Dr. Gregory, the physician of the Small-Pox Hospital, and author of the Report; and they believe that they cannot meet the wishes of the Right Honourable Secretary, for information on the subject, and for a refutation of the statement (if it could be refuted), better than by subjoining the questions put to Dr. Gregory, and the answers given by him. They were as follows:—

Q. When a person has been vaccinated successfully and effectually, do you not expect to find a cicatrix indicative of this in the arm?—A. Most certainly.

Q. Will you describe the character of the cicatrix which marks a perfect vaccination?—A. It should be very distinctly defined, perfectly circular, with indentations, and of a size not larger than that of a small wafer, or a sixpence.

Q. Did this characteristic mark of a perfect vaccination appear in the arm of John Richardson?—A. Certainly not; and, with regard to all the rest of the twelve, excepting William Johnson, the characteristic mark was wanting.

Q. Then you would have been justified in concluding that their vaccination had been imperfect and ineffectual, such as could not protect them against small-pox at any subsequent period of their lives?—A. Such marks ought not to have been received as evidence of the peculiar protection of vaccination.

Q. In fact, they might as well not have been vaccinated at all?—A. I believe as well not vaccinated at all.

Q. Have you any other proofs to state of their having been vaccinated previously to their taking the small-pox, of which they died?—A. No other distinct proofs. They all, meaning the twelve persons, had the persuasion that they had been vaccinated.

I am, &c.

HENRY HALFORD.

*January 25th, 1826.*

It is, of course, in reference to the above that we have received the following letter:—

*To the Editors of the London Medical and Physical Journal.*

GENTLEMEN,—Among the papers relative to vaccination, ordered to be printed by the House of Commons, is a letter from Sir Henry Halford, to Henry Hobhouse, Esq., containing certain queries put to me by the National Vaccine Board, and my answers thereto. By the introductory remarks, it would appear as if these answers were considered by Sir Henry as "a refutation of the statement" which I made in the last Annual Report to the Governors of the Small-Pox Hospital. I beg permission to state, through the medium of your widely-circulated Journal, that those answers are not considered by myself as refuting, in any degree, any of the statements made in my late Report, but merely as expressing in other words my opinions on the subject of imperfect vaccination. As the substance of my Report appeared in your Journal for February last, your readers, by a comparison of that with

Sir Henry Halford's letter, have an obvious means of judging for themselves.

I remain your very obedient servant,

GEORGE GREGORY.

*8, Upper John-street, Golden-square;*
*March 22,* 1826.

13. *History of the Small-Pox as it appeared on board His Majesty's Ship Phaeton.*—

*Victualling Office, 15th October,* 1825.

GENTLEMEN,—Agreeably to the request contained in Mr. Barrow's letter of the 27th ultimo, I repaired to Portsmouth, and made a particular inquiry into all the circumstances connected with the late visitation of small-pox on board his Majesty's ship Phaeton, on her passage to America, and have likewise seen and examined every officer and man remaining in the ship who had suffered from the late attack. I have to submit the following Report to the Board thereupon.

The Phaeton sailed from Portsmouth in the beginning of June, and on the 16th of that month Thomas Witcher, the boatswain's yeoman, was attacked with a disease, which in a day or two showed itself to be small-pox. This man had been allowed a few days' leave of absence, to visit his friends before the ship sailed; and it was discovered, after his illness, that the small-pox was raging in the same house wherein he took up his abode. His disease proved very severe, and he died on the 29th June, in Funchall Roads, at the island of Madeira.

On the 4th July, the disease again made its appearance amongst the midshipmen, and, in the course of five or six days, there were no less than seven midshipmen and nine of the ship's company attacked.

All the young gentlemen had been vaccinated when children; and I ascertained, by personal examination in all but one case, that the cicatrices were most perfect, and I have no doubt that they had passed under the influence of the vaccine inoculation in a regular and satisfactory manner.

Of the nine belonging to the ship's company, three stated that they had undergone vaccination when young; two were vaccinated by the surgeon on board; and two stated that they had had the small-pox in the natural way. The remaining two were boys, who had been vaccinated by the surgeon a few weeks before leaving Portsmouth.

With respect to the first three who stated that they had undergone vaccination, I have to observe that, in the case of Charles Clements, I have very great doubt whether he has passed through this operation in such a manner as to give reasonable hope of security; and, as regards the two, viz. John Thomson and John Smith, I am fully convinced that they never underwent vaccination in a proper manner. The first of the three had a very indistinct and unsatisfactory mark on his arm; the other two had nothing whatever to show. There were likewise some anomalies attending the cases of the two last, which will be noticed hereafter.

The second two, J. Reid and J. Munns, were passing through the stages of vaccination when attacked. The attack of Reid took place on the sixth day after vaccination, and that of Munns on the fourteenth: Reid fell a victim to the disease, and Munns escaped with difficulty.

The cases of J. Vick and William Colerigg, the surgeon considered to have been second attacks of small-pox. Vick declared to the surgeon that he had passed through the disease in a natural way, and showed marks on different parts of his body, which satisfied that gentleman of the truth of his assertions; but, as he was covered with the marks of his recent attack when I visited the Phaeton, it was impossible for me to decide. Colerigg, who died on the eighth day of his illness, stated that he had passed through the disease (small-pox) on board the Salvador del Mundo, in 1811; and Mr. Crichton, the surgeon of the Phaeton, who was an assistant surgeon of the Salvador del Mundo at the period, informed me that both small-pox and measles certainly prevailed on board that ship at the time mentioned, but that he did not recollect the individual. I have examined the surgeon's journal, and also the books of Plymouth Hospital for that year, but the name of Colerigg does not appear in either.

Regarding the boys, I have to observe, that they had been recently vaccinated on board, and passed through the disease in a very mild manner.

With the intention, however, of placing before the Board a condensed view of the cases of these individuals, I have drawn up the accompanying table, which contains every thing necessary to illustrate the most interesting part of the question of vaccination.

Of the whole number attacked, ten appear to have been properly vaccinated; of these, seven were severe: all recovered. Of two who were under vaccination at the time of the attack, one died, and the other narrowly escaped. Of three who had neither been vaccinated nor had passed through the small-pox, one died; and in the cases of the other two, J. Thomson and William Smith, the symptoms were of an anomalous nature.

The case of Charles Clements was one of a very doubtful aspect as to vaccination, and he passed through the regular stages of small-pox in the usual manner. Of two who declared that they had had small-pox formerly, one died and the other recovered.

Though, upon a consideration of the foregoing facts, I am obliged to confess that vaccination does not afford all the protection from small-pox which had at one time been anticipated, still there can be no doubt that it has had a most beneficial effect in modifying the disease; yet it must also be allowed that the proportion of three severe cases in ten is a considerable number. It should, however, be taken into consideration that the weather was then exceedingly hot, the thermometer being about eighty degrees in the shade, which, under any circumstances, must have had the effect of increasing the febrile action.

(Signed) W. BURNETT.

*To the Board.*

[*For the Table, see next page.*]

| *Names, Ages, and Qualities.* | *Whether Vaccinated or not.* | *Date of Attack.* | *Eruption appeared.* | *Fever ceased.* | *Eruption began to decline.* | *Whether secondary Fever appeared.* | *Event.* | *Remarks.* |
|---|---|---|---|---|---|---|---|---|
| | | 1825. | | | | | 1325. | |
| Thomas Wicher . . . . . . . . . | not . . . . . . . . . . | 16 June | became confluent | | — | — | died 29th June. | |
| Mr. Moore, midshipman . . ætat. 16 | cicatrix said to be perfect | 4 July | 5 July | 9 July | 9 July | none | recovered. | |
| Mr. Sleigh, master's assistant, ætat. 20 | vaccinated; cicatrix very perfect | ditto | 8 July | — | 13 July | none | returned to duty 26th July. | |
| Mr. Busk, midshipman . . ætat. 22 | ditto . . . . . . . . | ditto | 7 July | ran its course in a few days | | | do. 20th July. | |
| Mr. Lee, midshipman . . . . . . | this case was the same as Mr. Busk's. | | | | | | | |
| Mr. Warden, midshipman . ætat. 19 | vaccinated; and cicatrix perfect | ditto | 8 July | & became confluent | | appeared | do. 30th July. | |
| John Reid, A.B. . . . . . ætat. 19 | vaccinated on board, 24th June | ditto | 9 July | — | — | — | died 30th July | This man was indisposed on 1st July, apparently from his vaccine pustule; on 3d, he was well. He suffered much from an old pulmonic complaint during his illness. |
| J. Massie, midshipman . . ætat. 16 | vaccinated; cicatrix perfect | 7 July | eruption very trifling | — | — | none | recovered | Many of the pustules did not arrive at suppuration, though the eruptive fever was brisk. |
| John Harvey, mate . . . . ætat. 23 | ditto . . . . . . . . . | ditto | 8 p.m. | 9 July | 16 July | none | recovered. | |
| Ch. Clements, captain after-guard, ætat. 35 | very doubtful . . . . | ditto | the eruption in this case went through the stages in a distinct manner | | | | recovered. | |
| William Colerigg, A.B. . . ætat. 28 | declared he had small-pox in 1811 | ditto | 8 July | became confluent | — | appeared | died 17th July | This man showed the surgeon marks having the appearance of a former attack of small-pox. |
| T. Avenall . . . . . . . . ætat. 13<br>J. Sutton . . . . . . . . ætat. 12 | vaccinated in May; cicatrices perfect | ditto | 10 July | completed the stages in a very mild manner | | ditto | | |
| J. Thomson, seaman . . . ætat. 20 | not . . . . . . . . . . | ditto | no pustules appeared. | — | — | none | recovered | This man had no pustular eruption, but had an efflorescence, which on its disappearance left dry scales on the surface of the skin. |
| J. Vick . . . . . . . . . ætat. 30 | declared he had had the small-pox | ditto | 9 July | 12 July | 17 July | none | recovered | This man showed the surgeon satisfactory marks of a former attack of small-pox. |
| J. Munns, seaman . . . . ætat. 27 | vaccinated on board, 24th June | 8 July | ditto | 19 July | 20 July | appeared | recovered | |
| John Smith, seaman . . . ætat. 20 | not . . . . . . . . . | ditto | 10 July | 10 July | — | none | recovered | The eruption in this case had become pustular. He returned to duty 20th July. |
| J. Pritchard, under-butler to the Right Hon. Mr. Vaughan . . . . . . | vaccinated . . . . . | 3 Aug. | 5 August | 9 August | — | slight on 15th | Baltimore hospital; 26th, doing well. | |

### SURGERY.

14. ***On the Application of the Actual Cautery in the Erysipelas following Wounds.***—" The actual cautery, (says M. Larrey,) applied to the points reddest and nearest the wound, stops instantly the progress of the inflammation. This application, which causes scarcely any pain, is immediately followed by a gaseous effluvium, of an animal odour, rendered visible by a slight smoke which surrounds it, and by the disappearance of the redness, tense pain, and swelling of the parts. These cauterisations are not followed by suppuration, and are not capable of producing gangrene, as rubefacients are. The burnt parts of the cutis fall off in little charred scales, leaving no sensible cicatrix. The flow of pus from the wound, the suppression of which had preceded the erysipelas, is almost immediately restored; the strength of the patient increases; the weakened functions of the viscera, particularly the stomach, are strengthened, and thus concur in bringing the disease to a resolution."

M. Larrey does not attempt to explain the effects of the cautery; he only wishes to draw the attention of practitioners to the fact: he accounts, however, for some of the effects of the actual cautery. Its causing no sensible pain, he believes to arise from a coating, more or less thick, of a network of vessels, injected and covered with the epidermis already disorganised: which coating, he thinks, isolates and protects the nervous tissue of the skin. The burns do not suppurate, because the cautery has equally spared the sensible tissue of the skin, in which are found the only arterial vessels susceptible of producing suppuration; and, the skin itself not being broken, no cicatrix is left.

He relates two cases where the erysipelas, and adynamic fevers which accompanied it, disappeared, as if by enchantment. In the first case, six days after a contused wound of the right leg, the edges of the wound took on an erysipelatous character, and a general nervous affection showed itself. The erysipelas enlarged, and soon covered the whole limb and foot, which swelled. On the eleventh day, well-marked red lines stretched up the thigh, and little gangrenous spots showed themselves around the wound. The sensibility of the limb was considerably diminished: it was much puffed, and of a reddish violet colour. There were present nausea, vertigo, and much disturbance from the weight and tightness of the limb; pulse small and feverish; tongue black and dry; eyes dull and tearful; urine in small quantity, and black. A vomit was given, and some diluents; the limb wrapped in compresses, moistened with camphorated vinegar. At the next visit, finding the erysipelas in the same state, and the patient in much danger, it was decided to use the actual cautery to all the principal points of the erysipelatous swelling; beginning by the red bands on the thigh, then gradually coming to the knee, and leg, and foot, taking care to leave considerable distances between the cauterised points, and to end where the skin was adherent to the bony projections. Fifty little knobs of red hot iron were thus applied in less than ten seconds; and a moxa was used on the epigastrium. Compresses, with the camphorated vinegar, were applied. Low diet. At the next visit, the erysipelas was almost entirely gone, and the swelling of the limb considerably reduced; good suppuration was established in the wound; tongue had become moist, and cleaner; and the patient was so much improved, that, though not able to speak on the previous night, he now loudly said he was better. The same day he had some dark and extremely fetid alvine evacuations. In less than three days, all symptoms of the erysipelas had disappeared; and shortly after the patient left the hospital. The burnings were not followed by suppuration, and no cicatrices were left.

The other case was an erysipelatous affection of the scalp, where the application of the cautery was equally advantageous. *(Revue Med.)*

15. *Extirpation of the Neck of the Uterus.*—M. LISFRANC gave an account, at a late meeting of the Royal Academy of Medicine, of a case in which he had lately performed the above operation. The part was greatly tumefied, and in a carcinomatous condition. Hemorrhage, not sufficiently violent to call for any extraordinary measures, persisted for some days, and probably prevented the occurrence of any other accidents. The woman is, in fact, in complete convalescence. *(Archives Generales.)*

---

### CHEMISTRY.

16. *Test of the Presence of Organic Exhalations in the Atmosphere.*—Il Signor BIZIO having noticed the powerful effect exerted by anhydrous sulphuric acid in vapour over organic exhalations in the air, and the consequent production of black carbonaceous spots, has proposed the use of it for this purpose, and has dignified a little glass instrument, in which a portion of air is exposed to the vapours of the acid, by the name of the *diaftoroscopio.* He states that he has experimented frequently with the air of his laboratory, but never found it free from these kinds of exhalations.

By putting different substances into the air operated upon,—as, for instance, putrid matter, alcohol, essential oils, camphor, ether, odorous resins, &c.—a saturated atmosphere was procured, and results, in some degree comparative, obtained. The vapour of alcohol gave the most carbonaceous indication, and after it camphor. Ether gave no indications above that of the air containing it. *(Giornale di Fisica.)*

17. *Preservation of Lemon or Lime Juice.*—Lemon or lime juice, according to the experiments of Captain BAGNOLD, may be preserved without the addition of rum, spirit, or any other substance, by the process well known and practised for the preserving of green gooseberries and other fruits for domestic purposes. Lime juice was expressed from the fruit in Jamaica, in September 1823, strained, put into quart bottles, and carefully corked; these being put into a pan of cold water, were gradually raised to the boiling point; they were retained at that point for half an hour, and then allowed to cool. A bottle opened in April, 1824, was found to contain the juice in the state of a whitish turbid liquor, with the acidity and much of the flavour of the lime, nor did it appear to have undergone any alteration. The same juice, again bottled and heated, was set aside till March, 1825, when, upon examination, it was found in good condition, retaining much of the flavour of the recent juice. *(Trans. Soc. Arts.)*

MEDICAL POLICE.

18. ***On Chemists and Druggists prescribing for Patients.***—An inquest was lately held at Southampton, on the body of a female infant, named Louisa Mags. It appeared that the child had been under the care of Mr. Palk, who was only a druggist, and according to Act of Parliament not entitled to practise; and therefore his attendance could not be considered as that of a medical man. Mr. Keele, a surgeon, who had examined the body, stated that the child had died of general debility; there was no appearance of inflammation or acute disease, excepting in the liver, which was enlarged. The mother of the child had taken it to Mr. Palk, the druggist, who had ordered it some borax for the mouth, with some magnesia and castor oil. She did not apply for farther advice because she thought the medicine given by the druggist would do the child good;—she farther stated, that Mr. Palk did not say there was any danger of the child dying, but, on the contrary, that it would probably live fourteen or fifteen months.

The Coroner, in charging the Jury, made the following observations: —The parents of the child, it appeared, had applied for a parish coffin, which was refused them, (in consequence of the mother having stated that the child had received no medical advice,) till an enquiry had been instituted. He (the Coroner) had received two messages on the subject previous to calling this inquest; and, as he found the child had been attended only by Mr. Palk, he had requested Mr. Keele to open the body. He then directed their attention to the evidence of Mr. Keele, who had stated that the child's disease was hereditary: this he (the Coroner) believed—the ulceration of the mouth shewed it to be so; but Mr. Palk had stated the child's disease to be the thrush. He then pointed out the discrepancy in the evidence of the mother of the child and Mr. Palk. The latter said the child was dying when it was brought to him; but to the mother he gave his opinion directly contrary—he told her the child might live fourteen or fifteen months: this you will consider in returning your verdict. That the child died by the visitation of God there can be no doubt; but the question is, whether the child's life could not have been saved, or at any rate prolonged, and its sufferings mitigated, if it had received proper attention. And here he would observe, that Mr. Palk would have done but justice to the parents, if he had recommended them to take it to a regular practitioner; for it was not to be supposed that Mr. Palk was capable of rendering it that service which it required. Mr. Palk derived his knowledge only from behind a chemist's counter, and has not received sufficient medical education to enable him to undertake this or any other case of a medical nature. He therefore should not be allowed to practise, particularly in cases where men of tried experience, education, and ability, and with which the town abounds, were indispensably necessary, as in the present instance. "I should consider it a dereliction from my duty, as Coroner, if in future I did not institute judicial enquiry upon all cases where a party dies who has not had medical assistance from a person duly qualified to practise, agreeably to an act of parliament passed in the late king's reign, for the better regulating the practice of apothecaries in England and Wales."

The Jury, after some hesitation, brought in a verdict, "That the deceased died by the visitation of God, but that its death was hastened for the want of earlier and proper medical advice."

Our object in publishing this case is merely to remind our readers of the illegality of chemists and druggists *prescribing*.

---

MISCELLANEOUS.

19. ***Regulations respecting the Medical Studies required for Admission into the Medical Service of the Navy.***

*Victualling Office, 23d February,* 1826.

The Right Honourable the Lords Commissioners of the Admiralty having been pleased to direct, "that no person be admitted to be a candidate for situation of assistant surgeon in the royal navy, who shall not produce a certificate from one of the Royal Colleges of Surgeons of London, Edinburgh, and Dublin, of his fitness for that office; nor for that of surgeon, unless he shall produce a diploma, or certificate, from one of the said royal colleges, founded on an examination to be passed subsequently to his appointment of assistant surgeon, as to the candidate's fitness for the situation of surgeon in the navy; and that in every case the candidate producing such certificate, or diploma, shall also undergo a further examination before the medical commissioners of the Victualling Board, touching his qualifications in all the necessary branches and points of medicine and surgery for each of the steps in the naval medical service;' the Commissioners for victualling his Majesty's Navy, &c., do hereby signify, for the information of those persons to whom it may relate, that these regulations and directions will be strictly adhered to in future; and further, that previously to the admission of assistant surgeons into the navy, it will be required that they should have received a classical education, and possess in particular a competent knowledge of Latin; also,

That they should have served an apprenticeship, or have been employed in an apothecary's shop for not less than two years.

That their age should not be less than twenty years, nor more than twenty-six years.

That they should have attended an hospital in London, Edinburgh, Dublin, or Glasgow, for twelve months; and

That they should have attended lectures, &c. on the following subjects, for periods not less than hereunder stated, viz.

| | |
|---|---|
| Anatomy . . . | 18 months. |
| Surgery . . . | 18 ditto. |
| Theory of medicine . . | 12 ditto. |
| Practice of ditto . . | 12 ditto. |
| Chemistry . . . | 6 ditto. |
| Materia medica . . | 6 ditto. |
| Midwifery . . . | 6 ditto. |
| Actual dissections of the human body | 6 ditto. |

Although the above are the only qualifications which are absolutely required in candidates for the appointment of assistant surgeon, a preference will be given to those who, by possessing a knowledge of diseases of the eye, and of any branch of science connected with the profession, such as botany, medical jurisprudence, natural philosophy, &c. appear to be more peculiarly eligible for admission into the service.

It is also to be observed, that, by the rules of the service, no assistant surgeon can be promoted to the rank of surgeon until he shall have served full three years in the former capacity; and the Board have resolved that not any diploma or certificate of examination from either of the aforesaid royal colleges, shall be admitted towards the qualification for surgeon, unless the diploma or certificate shall be obtained on an examination passed after a period of not less than three years' service as assistant surgeon. By command of the Board, &c.

---

### MONTHLY CATALOGUE OF MEDICAL BOOKS.

### METEOROLOGICAL JOURNAL,

*From March 20, to April 19, 1826, inclusive.*

By Messrs. William Harris and Co. Mathematical Instrument Makers, 50, Holborn, London.

| | Moon. | Rain gauge | Therm. | | | Barom. | | De Luc's Hyg. | | Wind. | | Atmospheric Variations. | | |
|---|---|---|---|---|---|---|---|---|---|---|---|---|---|---|
| | | | 9 A.M. | Max. | Min. | 9 A.M. | 10 P.M. | 9 A.M. | 10 P.M. | 9 A.M. | 10 P.M. | 9 A.M. | 2 P.M. | 10 P.M. |
| Mar. | | | | | | | | | | | | | | |
| 20 | | | 44 | 48 | 36 | 29.84 | 29.85 | 73 | 74 | NW | N | Fine | Fine | Fine |
| 21 | | | 45 | 48 | 35 | 29.82 | 29.82 | 78 | 74 | NE | NEva. | Fair | | Cloud |
| 22 | | | 37 | 44 | 37 | 29.69 | 29.67 | 76 | 80 | NNE | N | Cloud. | | Fine |
| 23 | ○ | .39 | 40 | 42 | 33 | 29.49 | 29.34 | 79 | 87 | N | NE | Fine | Rain | Rain |
| 24 | | | 37 | 40 | 34 | 29.38 | 29.60 | 80 | 82 | ENE | NE | Cloud. | | |
| 25 | | | 38 | 42 | 34 | 29.62 | 29.59 | 67 | 76 | NE | NE | | Fine | Fine |
| 26 | | | 32 | 42 | 30 | 29.63 | 29.80 | 68 | 69 | NE | NE | Fine | Rain | |
| 27 | | | 36 | 42 | 34 | 29.82 | 29.82 | 66 | 61 | ENE | SW | | Fine | |
| 28 | | | 41 | 49 | 44 | 29.72 | 29.53 | 68 | 79 | SW | SW | | | |
| 29 | | | 48 | 49 | 31 | 29,47 | 29.66 | 73 | 69 | WNW | WNW | Cloud. | | |
| 30 | ☾ | | 39 | 47 | 32 | 29.92 | 30.10 | 66 | 60 | WNW | NW | Fine | | |
| 31 | | | 38 | 46 | 34 | 30.20 | 30.24 | 66 | 68 | W | W | | | |
| Apr. | | | | | | | | | | | | | | |
| 1 | | | 39 | 50 | 42 | 30.24 | 30.13 | 67 | 64 | W | W | | | |
| 2 | | | 45 | 52 | 50 | 29.99 | 30.01 | 66 | 81 | WSW | W | Fair | | |
| 3 | | | 55 | 66 | 45 | 30.02 | 30.09 | 76 | 75 | WNW | NW | Fine | | |
| 4 | | | 51 | 58 | 49 | 30.05 | 30 02 | 80 | 70 | WSW | W | | | |
| 5 | | | 51 | 57 | 48 | 29.98 | 29.97 | 74 | 63 | W | WSW | | | |
| 6 | | | 54 | 60 | 49 | 30.10 | 29.96 | 76 | 74 | WSW | WSW | | | |
| 7 | ● | | 54 | 63 | 50 | 29.96 | 30.06 | 77 | 68 | W | WSW | | | |
| 8 | | | 56 | 63 | 52 | 30.03 | 29.86 | 68 | 66 | W | SW | | | |
| 9 | | | 60 | 65 | 42 | 29.64 | 29.66 | 61 | 80 | SSE | WSW | | Rain | |
| 10 | | | 51 | 62 | 49 | 29.77 | 29.82 | 68 | 74 | W | SW | | Fine | Fair |
| 11 | | | 56 | 60 | 46 | 29.67 | 29.62 | 79 | 72 | WSW | SW | Rain | Rain | Rain |
| 12 | | | 51 | 54 | 45 | 29.07 | 29.46 | 79 | 75 | WSW | WNW | | | Fair |
| 13 | | .51 | 47 | 57 | 49 | 29.95 | 30.00 | 70 | 73 | NNW | WSW | Fair | Fine | Fine |
| 14 | | | 55 | 61 | 50 | 30.12 | 30.13 | 73 | 74 | W | WSW | | | |
| 15 | ☽ | | 59 | 59 | 50 | 30.12 | 30.05 | 75 | 73 | WSW | W | Fine | | Fair |
| 16 | | | 54 | 60 | 41 | 30.04 | 30.13 | 74 | 70 | NNE | N | Rain | | |
| 17 | | | 51 | 54 | 45 | 30.15 | 30.13 | 63 | 63 | NNW | SSE | Fine | | Fine |
| 18 | | | 48 | 58 | 43 | 30.05 | 30 03 | 68 | 61 | S | SSE | Foggy | | |
| 19 | | | 56 | 60 | 42 | 29.97 | 29.89 | 60 | 62 | SSW | ENE | Fine | | |

The quantity of rain fallen in the month of March, was 1 inch and 33.100ths.

